# Hybrid Models for Endoscopy Image Analysis for Early Detection of Gastrointestinal Diseases Based on Fused Features

**DOI:** 10.3390/diagnostics13101758

**Published:** 2023-05-16

**Authors:** Ibrahim Abdulrab Ahmed, Ebrahim Mohammed Senan, Hamzeh Salameh Ahmad Shatnawi

**Affiliations:** 1 Computer Department, Applied College, Najran University, Najran 66462, Saudi Arabia; hsshatnawi@nu.edu.sa; 2 Department of Artificial Intelligence, Faculty of Computer Science and Information Technology, Alrazi University, Sana’a, Yemen

**Keywords:** CNN, XGBoost, FFNN, fusion features, Kvasir dataset, gastrointestinal

## Abstract

The gastrointestinal system contains the upper and lower gastrointestinal tracts. The main tasks of the gastrointestinal system are to break down food and convert it into essential elements that the body can benefit from and expel waste in the form of feces. If any organ is affected, it does not work well, which affects the body. Many gastrointestinal diseases, such as infections, ulcers, and benign and malignant tumors, threaten human life. Endoscopy techniques are the gold standard for detecting infected parts within the organs of the gastrointestinal tract. Endoscopy techniques produce videos that are converted into thousands of frames that show the disease’s characteristics in only some frames. Therefore, this represents a challenge for doctors because it is a tedious task that requires time, effort, and experience. Computer-assisted automated diagnostic techniques help achieve effective diagnosis to help doctors identify the disease and give the patient the appropriate treatment. In this study, many efficient methodologies for analyzing endoscopy images for diagnosing gastrointestinal diseases were developed for the Kvasir dataset. The Kvasir dataset was classified by three pre-trained models: GoogLeNet, MobileNet, and DenseNet121. The images were optimized, and the gradient vector flow (GVF) algorithm was applied to segment the regions of interest (ROIs), isolating them from healthy regions and saving the endoscopy images as Kvasir-ROI. The Kvasir-ROI dataset was classified by the three pre-trained GoogLeNet, MobileNet, and DenseNet121 models. Hybrid methodologies (CNN–FFNN and CNN–XGBoost) were developed based on the GVF algorithm and achieved promising results for diagnosing disease based on endoscopy images of gastroenterology. The last methodology is based on fused CNN models and their classification by FFNN and XGBoost networks. The hybrid methodology based on the fused CNN features, called GoogLeNet–MobileNet–DenseNet121–XGBoost, achieved an AUC of 97.54%, accuracy of 97.25%, sensitivity of 96.86%, precision of 97.25%, and specificity of 99.48%.

## 1. Introduction

The gastrointestinal system consists of internal body organs. The system is divided into the upper system, which includes the mouth cavity, esophagus, and stomach, and the lower system, which contains the small intestine, colon, and rectum. The primary task of the gastrointestinal system is to break down food into essential elements for the body and to expel waste in the form of feces [1]. If any organ is disturbed, its functions do not work well, which affects the body. Many gastrointestinal disorders exist, such as infections, ulcers, polyps, and malignant tumors [2]. Cancers of the stomach, esophagus, colon, and rectum constitute about 3 million new cancer cases per year, and about 2 million people die annually from these diseases [3]. Malignant tumors in the upper and lower gastrointestinal tract threaten human life. For the upper gastrointestinal tract, esophageal cancer is the least prevalent, whereas stomach cancer is the fifth most common type of cancer and the third most common cause of death [4]. In contrast, malignant tumors of the lower gastrointestinal tract are the second most common cause of death and the third most common type of cancer [5]. There are many imaging techniques for the organs of the gastrointestinal tract. Wireless capsule endoscopy (WCE) [6] is a wireless capsule-sized camera that is swallowed through the mouth and takes thousands of images, starting from the pharynx until it reaches the rectum. This technique is good because it reaches all places of the gastrointestinal tract, such as the small intestine, but it takes two days to pass to the end of the gastrointestinal system. However, there are some problems with WCE, the device can be difficult to swallow and pass normally and can get stuck in the narrow places in the intestines and cause an obstruction. Thus, endoscopy is the gold standard for detecting diseases of the gastrointestinal system [7]. An upper GI endoscopy examines the stomach, esophagus, and the beginning of the small intestine. In contrast, a lower gastrointestinal endoscopy covers the colon and rectum. Endoscopy is a video to discover the gastrointestinal internal parts using high-resolution digital endoscopes [8]. Endoscopic examinations require expensive equipment and a skilled specialist. Detection by endoscopy is imperative for colorectal cancer to be removed. Therefore, detecting benign tumors is necessary to remove them before they develop into a malignant type. However, the diagnostic ability of endoscopy varies according to the experience of doctors, which may result in colon and rectal cancer. Accurate diagnosis of diseases is vital to avoid malignancy and to survive. Esophageal infections are a normal and pathological condition, and early detection of esophagitis is necessary before complications lead to ulcers, stenosis, and bleeding [9]. If left untreated it develops into cancer. Therefore, a distinction must be made between the esophagus and the z-line. Endoscopy technology produces a video that converts to thousands of frames, increasing the burden of the doctors to keep track of all the video frames. The frames appear healthy; the disease (lesion, ulcer, or bleeding) does not occur except in a few frames. Therefore, this technique requires examining all frames, which takes a long time and is a tedious process [10]. Computer-aided diagnostic methods are used to support clinicians and experts in addressing the challenge of manual insufficiency. With the advancement in AI technologies, many researchers have focused on its application in healthcare, including biomedical image processing. Artificial intelligence techniques are used to detect gastrointestinal diseases and develop systems that can detect infections, ulcers, bleeding, polyps, colon cancer, and stomach cancer. The application of automated techniques is expected to improve the efficiency of diagnosis, reduce the gap between the number of doctors and patients, and reduce diagnostic costs. Many researchers have proposed deep learning techniques for analyzing and diagnosing medical images, including CNN models. CNN models are a good research topic in medical imaging diagnostics to help radiologists and medical specialists provide high-quality healthcare. CNN is an integrated model with dozens of layers to analyze thousands of endoscopy images and extract accurate feature maps to detect infections, ulcers, bleeding, polyps, and colorectal cancer. In this study, several effective systems for diagnosing endoscopy images of the gastrointestinal tract were developed. Due to the similarity of the clinical signs between infections and ulcers and the need for early detection of polyps, this study focused on developing hybrid techniques between deep and machine learning. CNN models have superior capabilities to extract features that are not visible to the naked eye, thus extracting features from many CNNs and integrating feature maps vectors CNN.

Artificial intelligence (AI) has made significant contributions to medical image processing, revolutionizing the field with its ability to analyze and interpret complex medical images. Some of the most important applications of AI in this domain include:

Classification of medical images: AI algorithms can analyze medical images such as endoscopy, X-rays, CT scans, and MRI scans to classify them into different categories, which helps in detecting diseases and abnormalities. For example, AI can identify cancerous tumors or diagnose certain conditions such as gastrointestinal diseases, pneumonia or retinal diseases, heart diseases, and skin diseases.

Computer-Aided Detection/Diagnosis (CAD): AI-based CAD systems can act as a “second opinion” by flagging potential abnormalities in medical images. These systems assist radiologists in the detection of tumors, lesions, or other abnormalities that may be missed or difficult to spot. CAD systems help reduce human error and improve diagnostic accuracy.

Segmentation and Annotation: AI techniques can segment medical images, separating different structures or organs for further analysis. This enables precise measurements and quantitative analysis. AI can also assist in annotating medical images, marking specific regions of interest for radiologists or clinicians.

Disease Detection and Diagnosis: AI algorithms assist in detecting diseases and conditions by analyzing medical images. They can learn patterns and features associated with various diseases, aiding in early detection and accurate diagnosis. For example, AI could help identify signs of gastrointestinal disease in endoscopic images, Alzheimer’s disease in brain scans, or detect diabetic retinopathy in retinal images.

Image Reconstruction and Enhancement: AI can reconstruct or enhance medical images to improve their quality or extract additional information. This can be particularly useful when dealing with noisy or low-resolution images. AI algorithms can fill in missing data or enhance image details, assisting radiologists, endoscopists, and physicians in making informed decisions.

Prognosis and Predictive Analytics: AI can analyze medical images to predict patient outcomes and provide prognostic information. By identifying patterns and features in images, AI algorithms can estimate disease progression, survival rates, or response to treatment, aiding in personalized patient care.

These applications of AI in medical image processing have the potential to enhance diagnostic accuracy, improve patient outcomes, and streamline healthcare workflows by reducing the time and effort required for manual analysis. However, it is important to note that AI systems should always be used in conjunction with human expertise and not as a replacement for clinical decision making.

The main contribution of this study is as follows:

- Classification of endoscopy images of gastrointestinal diseases by a hybrid methodology CNN-FFNN and CNN-XGBoost based on the GVF segmentation algorithm.
- Fusion of features of CNN models and their classification by FFNN and XGBoost based on the GVF segmentation algorithm.

The rest of this paper is organized as follows: Section 2 discusses relevant techniques and results from the literature. Section 3 explains the methodologies and tools applied to analyze endoscopy images from gastroenterology. Section 4 presents the results of the proposed methodologies. Section 5 discusses the performance of the methodologies and compares their results. Section 6 concludes the study.

## 2. Related Work

Öztürk et al. [11] developed three CNN models for classifying gastroenterological endoscopy images based on combining LSTM layers. The features are extracted from each CNN model and sent to an LSTM layer to evaluate the contribution to a dataset with various image counts. Imran et al. [12] propose a DCNN architecture with multiple pathways and layers to improve the efficiency of detecting endoscopy image abnormalities of the human gastrointestinal tract. The network works on two tracks with different resolutions, each with four units. Different degrees of image resolution may be useful in detecting gastrointestinal tract lesions. Yoshiok et al. [13] reported comparison of the performance of four CNN models to the analysis of endoscopy images to detect of esophagitis. The GoogLeNet achieved an F1-score that was better than the other models, whereas MobileNet V3 achieved a better average positive rate. Zahra et al. [14] presented a system for detecting abnormalities in WCE images of the gastrointestinal tract. The images were enhanced, the region of interest was extracted by the thresholding method, and the color, shape, and texture features were extracted. Finally, the features were fed to SVM to classify the extracted features. Shima et al. [15] described a method for detecting the anatomical features of endoscopy images of the gastrointestinal tract by supervised CNN and their comparison with semi-supervised CNN. The results showed that the supervised CNN achieved better results than the semi-supervised CNN. Ibtesam et al. [16] studied the retraining of five pre-trained CNN models to classify a Kvasir dataset containing eight classes, including an anatomical parameter, disease condition, and medical procedure. Inception-v3 and ResNet achieved 90% and 97.6% accuracy. Xing et al. [17] reported on a hybrid learning framework for tagging in medical endoscopy images and its fusion with deep learning. Experiments demonstrated the superiority of the framework for classifying the Hyper-Kvasir dataset with an accuracy of 95%. Ranit et al. [18] proposed a lightweight Mobile-PolypNet for the segmentation of colorectal polyps. The network uses a set of blocks of the autoencoder to improve the system’s efficiency. The network was trained and tested on the Kvasir dataset, achieving a DICE score of 93.5%. Subhashree et al. [19] developed an intelligent system for detecting abnormalities in GI endoscopy images based on CNN and time–frequency analysis. The images were improved, and separate waveform coefficients were extracted and submitted to two CNN models for training and testing at two levels. The system achieved an accuracy of 93.75% in the first level. Ali et al. [20] used pre-trained CNNs with filters to improve endoscopy images to detect GI disease. The preprocessing stage was combined with the classification methods of the Kvasir dataset, which achieved an accuracy of 90.17%. Debesh et al. [21] presented a classic U-Net architecture for hashing the Kvasir-Instrument dataset, which achieved a Jaccard index of 85.78% and a dice factor of 91.58%. Ramzan et al. [22] described a new framework for image preprocessing, integrating texture features, LBP, and deep learning serially to improve diagnostic imaging of gastrointestinal diseases. The improved features were selected by PCA and entropy. Yogapriya et al. [23] developed a model that integrates traditional algorithms and data augmentation with CNNs to classify gastrointestinal diseases. The model with the VGG16 network achieved better results than the rest of the CNN networks, achieving an accuracy of 96.33% and an F1 score of 96.5%. Muhammad et al. [24] reported on two deep learning models based on the hybrid crow–moth method for classifying gastrointestinal disease datasets. The features were extracted using average layers and then combined with crow–moth features. Muhammad et al. [25] used contrast enhancement technology to improve images and segmentation of the region of interest by saliency map, feeding regions of interest to MobileNet-V2 for training and feature extraction. The authors applied a hybrid whale method to select the best features.

The researchers devoted their efforts to developing a modern technique to obtain good results for diagnosing abnormalities in diseases of the gastrointestinal tract. The characteristics of gastrointestinal disorders are similar in the early stages, so this study focused on extracting the attributes from the region of interest from many methods and integrating them to achieve satisfactory results for the early detection of gastrointestinal diseases.

## 3. Materials and Methods

### 3.1. Description of the Kvasir Dataset

In this study, the performances of the systems were evaluated on endoscopy images from the available Kvasir dataset. Endoscopy images were obtained by the Vestre Viken Health Trust (VV) of four hospitals and the Cancer Registry of Norway (CRN), Norway. CRN provides knowledge about cancer to prevent its spread and is part of a regional body for Norway under the supervision of the Oslo Hospital. The Kvasir dataset images were classified into eight types divided into three anatomical landmarks (ALs), three types of pathological findings (PFs), and two types of polyps removal (PR). Endoscopy experts have classified and annotated all images. The dataset consists of 8000 images distributed evenly among eight classes. LA include three types: cecum and Z-line pylorus. LA within the gastrointestinal tract can be identified endoscopically as marker of inflammation and ulcers. In contrast, PFs include esophagitis, ulcerative colitis, and polyps. PFs are abnormal signs of the gastrointestinal tract that are identified endoscopically [26]. These clinical characteristics may be signs of an ongoing disease or an early cancer stage. PR includes two types: dyed and lifted polyps and dyed resection margins, which are polyps that may be a harbinger of cancer. All images of the Kvasir dataset are in RGB color with resolutions ranging from 720 × 576 to 1920 × 1072.

### 3.2. Enhancement of the Images of the Kvasir Dataset

When acquiring images through the endoscope, some artifacts negatively affect the image enhancement stages and lead to incorrect results. The artifacts comprise air bubbles, food residues in the upper digestive tract, stool residues in the lower digestive tract, mucous membranes, and fluids [27]. All these artifacts are considered features in the next stages, and they are artifacts and have nothing to do with the disease. Additionally, the low contrast between the borders of the disease and the healthy part presents a challenge to extract and analyze the diseased part by extracting its features. Thus, removing artifacts is essential for proper feature extraction, and increased low variance is necessary for efficient segmentation of a region of interest. This study calculated the mean achromatic area for each RGB channel. After that, an averaging filter was applied to improve the images and remove artifacts, followed by increasing the contrast of the low area between the infected and the healthy regions by a contrast-limited adaptive histogram equalization (CLAHE) method [28].

First, to refine the endoscopy images of the Kvasir dataset, each image was passed through an averaging filter with an operator of size 6 × 6. The image is processed pixel by pixel, called the target pixel, based on the value of 35 adjacent pixels. The filter continues to run until the last pixel in the image is processed, as in Equation (1) [29]:

(1) $$F\left(x\right)=\frac{1}{N}\overset{N-1}{\underset{i=0}{\sum }}a\left(x-i\right)$$

where *F*(*x*) means the output, *N* means the number of pixels, *a*(*x*) means the input, and *a*(*x* − *i*) means the prior input.

**Figure 1:**
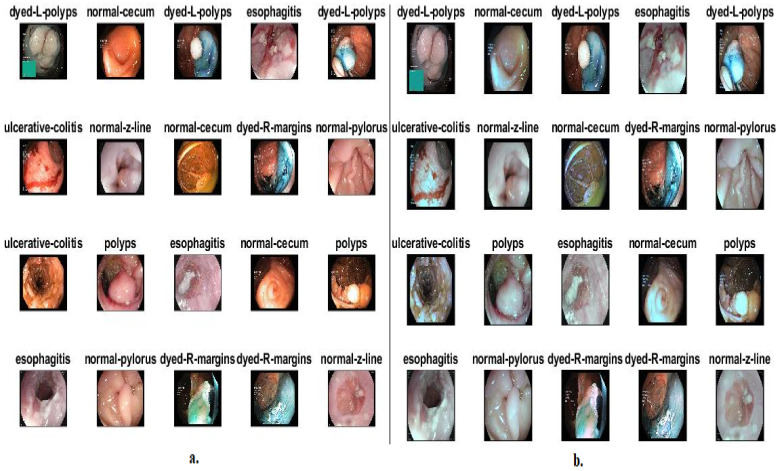
A sample from the Kvasir gastroenterology dataset classes: (**a**) before improving and (**b**) after improvement.

In order to address the low contrast between the infected and healthy areas, images are passed through a CLAHE method optimization method [30]. The method works to light up the dark areas by distributing bright pixels to the dark areas. The method processes pixels based on the value of adjacent pixels. When processing each pixel, the method compares it with the value of neighboring pixels as follows: if the value of the neighboring pixels is greater than the target pixel, then the contrast is increased, whereas the contrast decreases when the target pixel is larger than the adjacent ones. The method continues for each pixel of the image until the contrast of the affected areas is improved. Figure 1b shows a set of images from the Kvasir dataset after optimization.

### 3.3. Gradient Vector Flow Method

The GVF method is an extended method of the active contour method, which is defined by the following steps: first step, detect the edges of the affected regions *F*(*x*, *y*) from endoscopy images *I*(*x*, *y*). Equation (2) illustrates the mechanism for detecting the edges of grayscale images [31]. The GVF algorithm was used because it spatially extends the gradient vectors of the edge map, which produces a vector that contains information about the edges of the lesion throughout the image [32].

(2) $$f\left(x,y\right)=-|\nabla \left[G\sigma \left(x,y\right)*I\left(x,y\right)\right]{|}^{2}$$

where ∇ refers to gradient operator and *Gσ* (*x*, *y*) refer to a 2D Gaussian function with *σ* standard deviation.

**Figure 2:**
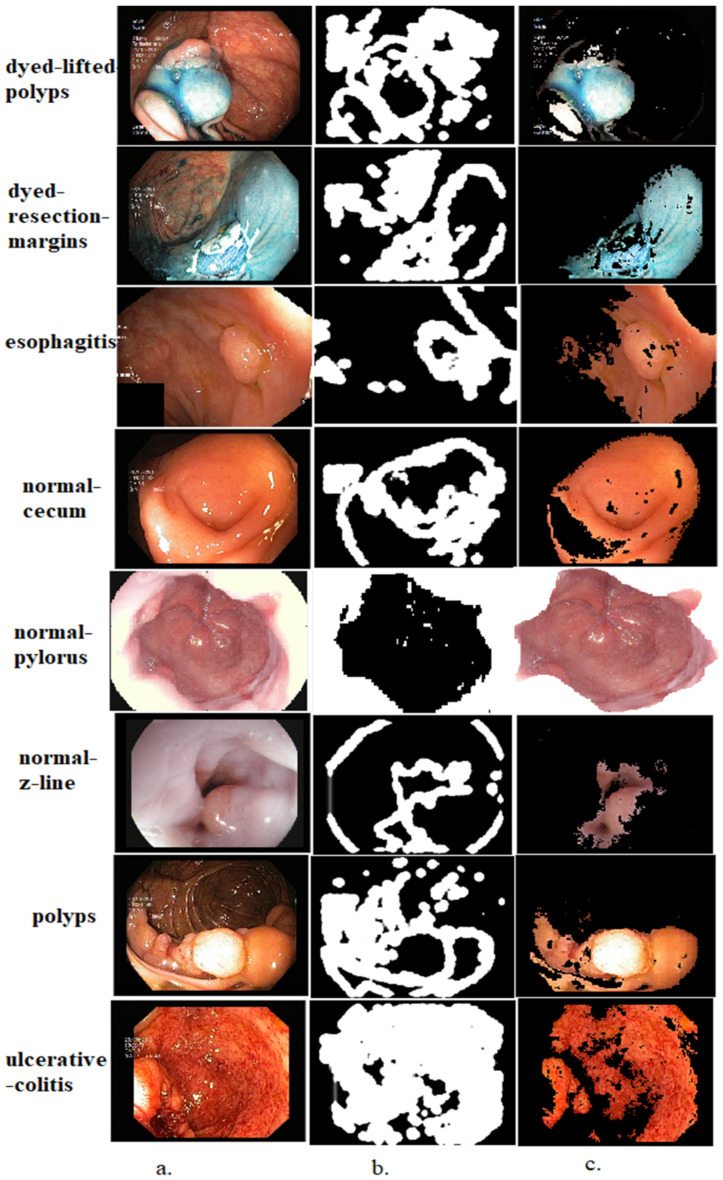
A sample of all classes from the Kvasir dataset after applying the GVF segmentation algorithm. (**a**) Original image; (**b**) image segmentation; (**c**) region of interest.

The flow minimizes the energy functionalization in the gradient vector by the GVF method. The functional energy depends on the smoothing and the level of edge noise present in the image. The image edge noise level is measured through the parameter μ. If the edge noise is high, the parameter μ should be increased, which leads to a decrease in the noise around the edges and a weakening of the contour, as in Equation (3) [33].

(3) $$\varepsilon =\iint \mu \left(u_{x}^{2}+v_{x}^{2}+v_{y}^{2}\right)+\left|\nabla f{|}^{2}\;\right|g-\nabla f{|}^{2}dxdy$$

where g refers to a gradient vector flow that can be derived from Euler equations that encourage edge map gradients that make g−∇f smaller. When the gradients are large, g−∇f is multiplied by the square of the gradient length subscripts that indicate partisan derivatives; $\nabla f=f_{x},f_{y}$ refers to a gradient map that shows edges. The affected area is segmented with specific parameters using gradient vector flow models. These models mark the edges of the affected regions and separate them from the rest of the image. The aim of applying this algorithm is to segment the infected areas and separate them from the healthy areas for further analysis. This method is an important contribution to this study as the affected areas of all the endoscopy images from the Kvasir dataset are segmented and saved in a new folder called Kvasir-ROI. CNN models receive endoscopy images of the gastrointestinal tract to the new Kvasir-ROI dataset. Thus, the CNN models work on analyzing endoscopy images of infected areas only instead of feeding them complete images including the healthy regions. Figure 2 shows a dataset sample that underwent segmentation and selected only affected areas.

### 3.4. CNN Models for the Extract Feature

CNN networks are characterized by their superior ability to process, interpret, and analyze the input data with high efficiency. The field of health care, especially the processing of biomedical images, has enjoyed a large share of CNN networks for analyzing medical images and diagnosing and predicting diseases and cancers at an early stage [34]. CNN is characterized by dozens of convolutional, pooling, fully connected, and other auxiliary layers. Convolution is a commutative operation involving two real-value functions. The input to a convolutional layer is a multidimensional array, and the filter is also a multidimensional array of parameters tuned by the learning process [35]. The convolutional layers receive endoscope images with an RGB color space of size *m* × *n* × *j*, where *m*, *n*, and *j* represent the width, height, and number of color channels of an image. Each CNN has a different number of convolutional layers. Each layer has a different filter corresponding to the image size regarding width, height, and number of filter channels. The filter *f* (*t*) wraps around a region of the image *x*(*t*) as in Equation (4). Each filter cell will be multiplied by the corresponding cell in the image to produce a matrix with smaller dimensions than the original image [36].

(4) $$y\left(t\right)=\left(x*f\right)\left(t\right)=\int x\left(a\right)f\left(t-a\right)\;da$$

where *f* (*t*), *x*(*t*), and *y*(*t*) denote the filter, input, and the image output, respectively.

Thus, a feature map is created by wrapping filters around the image. The number of feature maps equals the number of filters in the layer, and the layer’s output becomes the input for the next layer [37]. The convolutional layers are followed by a ReLU helper layer whose task is to pass only positive values, while converting negative values to zero, as in Equation (5).

(5) $$\mathrm{ReLU}\left(x\right)=\left\{\begin{array}{cc} x, & x\geq 0\\ 0, & x<0 \end{array}\right.$$

The output of convolutional layers is millions of neurons, connections, and parameters; therefore, networks will need complex calculations and a lot of training time. Thus, pooling layers will reduce the high aspect ratio, which reduces the image dimensions by pooling the output of a group of neurons in one layer to a single neuron in the next layer. There are two types of pooling layers. Either maximum or average grouping performs the calculations [38]. The Max pooling method uses the greatest value from a group of neurons and represents it with one neuron in the next layer, as in Equation (6), whereas the average pooling method calculates the average for a group of selected neurons and represents it with one neuron in the next layer, as in Equation (7) [39].

(6) $$z\left(i;\;j\right)=max_{m,n=1\ldots .k}\;f\left[\left(i-1\right)p+m;\;\left(\;j-1\right)p+n\right]$$

(7) $$z\left(i;\;j\right)=\frac{1}{k^{2}}\underset{m,n=1\ldots .k}{\sum }f\left[\left(i-1\right)p+m;\;\left(\;j-1\right)p+n\right]$$

where *f*, *p*, *k*, and *m*, *n* are the wrapped filter, p-step filter, number of pixels, and matrix sites.

The fully connected layers (FCLs) are the classification layers in CNNs. In these layers each neuron is connected to all neurons in the previous layer. The FCL transforms map features from high-dimensional to vector levels. The SoftMax function is non-linear and useful for handling classification problems. SoftMax is used with multiple classes; it divides the output between 0 and 1 according to the classes and then labels each image to its appropriate class [40].

Finally, in the last convolutional layer of the GoogLeNet, MobileNet, and DenseNet121 models are high-level features with sizes: (7, 7, 512), (7, 7, 1024), and (16, 32, 512), respectively. The global average pooling layer represents the high levels of features in feature vectors with sizes of 4069, 1024, and 1024 for the GoogLeNet, MobileNet, and DenseNet121 models, respectively. Therefore, the size of the gastrointestinal disease dataset is represented by feature matrices with sizes 8000 × 4096, 8000 × 1024, and 8000 × 8000 × 1024 for the GoogLeNet, MobileNet and DenseNet121 models, respectively.

### 3.5. Training and Performance Evaluation

The classification phase, including training and testing of the systems, is based on the previous phases of medical image processing. After enhancing the images, the ROI was segmented and the images were sent to CNN models to extract the features. Because of the high dimensions of the features, the CNN feature maps were passed to the PCA method [41] to select the representative features and delete the redundant and unimportant ones. [42] FFNN and XGBoost receive high-representation, low-dimensional features [43]. The classification phase includes an inductive phase called the training phase to build a taxonomic model. The deductive stage, the most important stage for the system’s efficiency, is called the testing stage.

#### 3.5.1. FFNN Network

**Figure 3:**
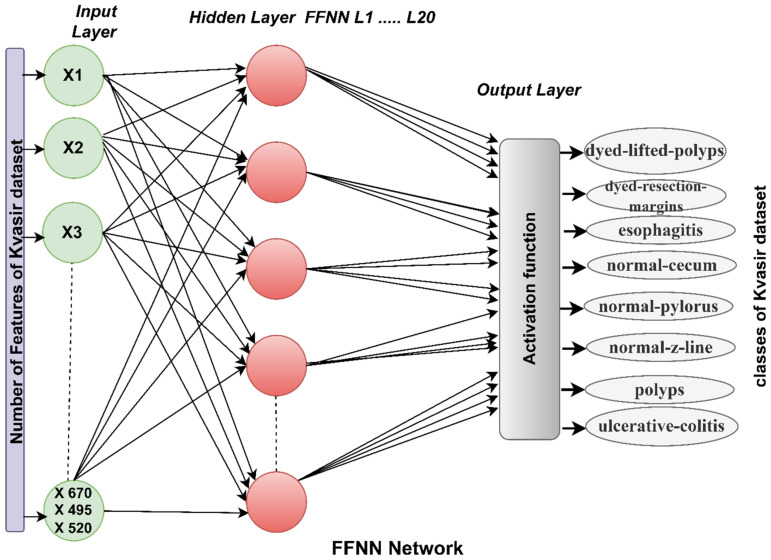
FFNN architecture for endoscopy image analysis of the Kvasir gastroenterology dataset classification.

FFNN has the ability to handle computational complexities and different parameters and perform classifications efficiently according to the training data. Whenever the training data is well trained, this leads to good results for the test data [44]. Training the network well depends on its internal structure, which consists of three basic layers: the input, hidden, and output layers. Increasing the hidden layers and neurons does not necessarily lead to satisfactory results; therefore, the network efficiency may improve when reducing the hidden layers and interconnected neurons. The number of hidden cells varies from one network to another. Thus, no fixed mechanism exists for identifying hidden layers and their associated neurons to obtain accurate classification results [45]. Thus, the number of hidden layers is adjusted through trial and error. The learning method is based on adjusting the weights continuously, returning from the output layers to the hidden layers and updating the weights. Each time the weights are updated, the minimum square error (MSE) is calculated between the actual $x_{j}$ and expected $y_{j}$ values, as in Equation (8). The network continues until no change appears with the least error value and the weights are fixed at the minimum error rate. For the network mechanism, the input layer receives features extracted from the CNN models (GoogLeNet, MobileNet and DenseNet121). The number of entries for FFNN is 670, 495, and 520, based on the features of GoogLeNet, MobileNet, and DenseNet121, respectively. Features move to hidden layers, where they are analyzed and interpreted through 20 hidden layers. Finally, the output layer shows the FFNN performance results [46]. The output layer consists of eight neurons, each representing a class in the dataset, where each image is mapped to an appropriate neuron (class). Figure 3 shows the basic structure of FFNN for receiving, analyzing, and interpreting features of the GoogLeNet, MobileNet, and DenseNet121 models.

(8) $$\mathrm{MSE}=\frac{1}{k}\overset{k}{\underset{j=1}{\sum }}\;(x_{j}-y_{j}{)}^{2}$$

where *k* is the number of data points.

#### 3.5.2. XGBoost Algorithm

XGBoost is a machine learning algorithm that works with the ensemble learning approach. A single model may not achieve good results in some algorithms, so the ensemble learning model offers a powerful approach to predicting multiple learners; the output is a single model. The algorithm sequentially generates many decision trees with different weights so that the algorithm merges weak learners with strong learners to produce an effective model; this process is called boosting. The boosting mechanism builds trees sequentially so that each subsequent tree takes advantage of the previous tree’s errors and reduces them [47]. Thus, any new tree is an updated version of the previous one with fewer errors than the previous one. In boosting, basic learners are weak learners who contribute some information vital to prediction. Thus, boosting can produce a strong learner by combining weak learners. When training the network, the weights of the variables that predict the decision tree are incorrectly increased. The variables are fed to the next tree to solve the errors in the previous tree, and so the network continues until the prediction of an effective and accurate dataset.

The first strategy is to classify endoscopy images from the Kvasir dataset by three pre-trained GoogLeNet, MobileNet, and DenseNet121 models. The second strategy is to analyze endoscopy images from the Kvasir gastroenterology dataset using the GoogLeNet, MobileNet, and DenseNet121 models based on the GVF segmentation algorithm. In this method, the images are improved and the infected areas are segmented and isolated from the healthy areas so that the CNN models receive the photos from the dataset after segmentation of the infected areas.

**Figure 4:**
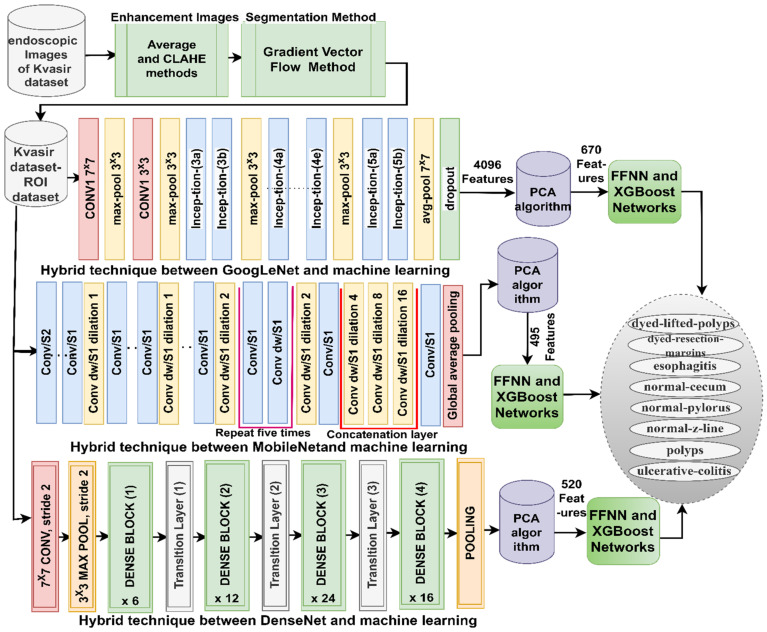
Hybrid methodology: CNN–FFNN and CNN–XGBoost based on the GVF segmentation algorithm for endoscopy image analysis to classify the Kvasir gastroenterology dataset.

The third strategy for analyzing endoscopy images from the Kvasir gastrointestinal disease dataset is by a hybrid technique; CNN–FFNN and CNN–RF based on the GVF and PCA algorithms goes through several stages (as shown in Figure 4): First, passing the endoscopy images to the averaging and CLAHE techniques to improve the images. Second, applying the GVF algorithm to segment the region of interest for further analysis in the following stages. Third, applying the GoogLeNet, MobileNet, and DenseNet121 models to analyze regions of interest by convolutional, auxiliary, and pooling layers. Feature maps of 8000 × 4096, 8000 × 1024, and 8000 × 8000 × 1024 are produced for the GoogLeNet, MobileNet, and DenseNet121 models, respectively. The fourth stage is to obtain more representative features for the affected and diseased regions and delete the repetitive features using the PCA method [48]. The PCA method produces reduced feature vectors organized into feature matrices of sizes 8000 × 670, 8000 × 495, 8000 × 520, and 8000 × 520 for the GoogLeNet, MobileNet, and DenseNet121 models, respectively. The fifth stage is the training phase and performance evaluation by FFNN and XGBoost. FFNN and XGBoost receive critical feature vectors after the PCA method [49] and divide them into two parts: 80% for training and validating a model and 20% for measuring its performance.

**Figure 5:**
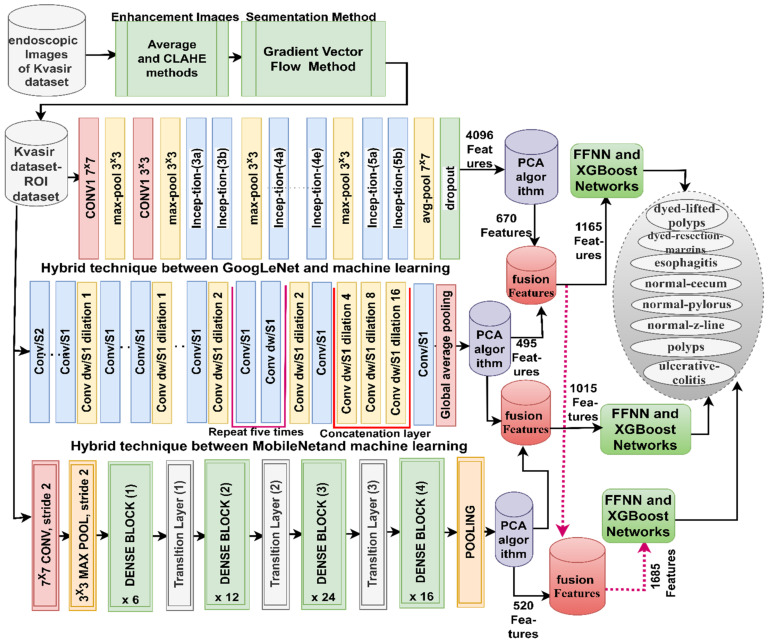
Hybrid methodology: CNN–FFNN and CNN–XGBoost based on fused CNN features and the GVF segmentation algorithm for endoscopy image analysis to classify the Kvasir gastroenterology dataset.

The fourth strategy for analyzing endoscopy images to detect gastrointestinal diseases is by a hybrid model based on fused CNN features and the GVF and PCA algorithms, as shown in Figure 5. The first four steps of the third strategy are the same in this strategy. Fifthly, the fusion between the CNN features are as follows: GoogLeNet–MobileNet, MobileNet–DenseNet121, GoogLeNet–DenseNet121, and GoogLeNet–MobileNet–DenseNet121. So, the feature matrices in this strategy become 8000 × 1165, 8000 × 1015, 8000 × 1165, and 8000 × 1685 for the fused models GoogLeNet–MobileNet, MobileNet–DenseNet121, GoogLeNet–DenseNet121, and GoogLeNet–MobileNet–DenseNet121, respectively. Sixth, the training phase and performance evaluation by FFNN and XGBoost. FFNN and XGBoost receive essential feature vectors after the PCA method and divide them into 80% for training and validating a model and 20% for measuring its performance.

## 4. Results of Systems

### 4.1. Split of the Kvasir Dataset

The model performance was measured on endoscopy images from the Kvasir gastroenterology dataset. The Kvasir dataset consists of 8000 images distributed evenly across eight classes. During the training phase, the dataset was divided into 640 images (64%) for systems training, 160 images (16%) for validation, and 200 images (20%) for testing.

### 4.2. Metrics of Models Evaluation

When implementing systems and testing their performance, a confusion matrix can be produced, which represents the gold standard for systems performance. A confusion matrix is in a form similar to a quadrilateral matrix. The columns represent the number of target samples, which corresponds to the number of output samples by the rows. All samples in the confusion matrix are correctly classified samples located in the main diagonal and are called true positives (TPs). At the same time, the other cells represent false positives (FPs) or false negative (FN) samples and are incorrectly classified. The results of the systems are measured through Equations (9)–(13) [50].

(9) $$\mathrm{AUC}=\frac{\mathrm{TP}\mathrm{Rate}}{\mathrm{FP}\mathrm{Rate}}*100\%$$

(10) $$\mathrm{Accuracy}=\frac{\mathrm{TN}+\mathrm{TP}}{\mathrm{TN}+\mathrm{TP}+\mathrm{FN}+\mathrm{FP}}*100\%$$

(11) $$\mathrm{Sensitivity}=\frac{\mathrm{TP}}{\mathrm{TP}+\mathrm{FN}}*100\%$$

(12) $$\mathrm{Precision}=\frac{\mathrm{TP}}{\mathrm{TP}+\mathrm{FP}}*100\%$$

(13) $$\mathrm{Specificity}=\frac{\mathrm{TN}}{\mathrm{TN}+\mathrm{FP}}*100\%$$

### 4.3. Results of CNN Models

This section discusses the performance of the pre-trained GoogLeNet, MobileNet, and DenseNet121 models. The pre-trained CNN models are based on an ImageNet dataset with over a million images of over a thousand classes. This huge dataset lacks medical images, so pre-trained CNN models do not reach good results for classifying some medical datasets. In this study, the experience gained from the CNN models to classify the ImageNet dataset has been transferred to perform the new task of classifying the Kvasir gastroenterology dataset. The input layers of the GoogLeNet, MobileNet, and DenseNet121 models receive endoscopy images of the Kvasir gastroenterology dataset and pass them to convolutional layers to represent the images in high-level feature maps. Pooling layers reduces high dimensionality and feature manipulation through helper layers. Fully connected layers receive feature maps and convert them from 2D to 1D. Finally, the SoftMax function adjusts each carrier to its appropriate class.

Table 1 summarize the results of the pre-trained GoogLeNet, MobileNet, and DenseNet121 models for endoscopy image classification of the Kvasir gastroenterology dataset. GoogLeNet achieved an AUC of 91.46%, accuracy of 88.2%, sensitivity of 88.05%, precision of 88.34%, and specificity of 98.19%. The MobileNet achieved an AUC of 90.99%, accuracy of 86.4%, sensitivity of 86.34%, precision of 86.34%, and specificity of 97.96%. The DenseNet121 achieved an AUC of 89.13%, accuracy of 85.3%, sensitivity of 85.23%, precision of 85.29%, and specificity of 97.85%.

### 4.4. Results of CNN Models Based on the GVF Algorithm

This section discusses the performance of the GoogLeNet, MobileNet, and Dense-Net121 models based on the GVF hashing algorithm. In this section, the endoscopic images are improved by an average filter and then passed to the CLAHE method to highlight the edges of the lesion. Then, infected regions are divided and separated from healthy regions and saved in a folder named Kvasir-ROI. The models receive pre-trained GoogLeNet, MobileNet, and DenseNet121. Regions of interest are analyzed through convolutional layers and pooling and saved in deep feature maps. A fully connected layer categorizes the feature maps.

Table 2 summarize the results of the pre-trained GoogLeNet, MobileNet, and DenseNet121 models based on the GVF algorithm for endoscopy image classification of the Kvasir gastroenterology dataset. The GoogLeNet–GVF achieved an AUC of 91.8%, accuracy of 90.8%, sensitivity of 91.09%, precision of 90.81%, and specificity of 98.49%. The MobileNet–GVF achieved an AUC of 91.84%, accuracy of 91.5%, sensitivity of 91.56%, precision of 91.55%, and specificity of 98.83%. The DenseNet121–GVF achieved an AUC of 90.89%, accuracy of 90.1%, sensitivity of 89.83%, precision of 90.03%, and specificity of 98.35%.

### 4.5. Results of Hybrid Models of CNN, FFNN, and XGBoost

The section summarizes the results of the hybrid methodology between CNN models (GoogLeNet, MobileNet, and DenseNet121) and the FFNN or XGBoost networks for endoscopy image classification of the Kvasir-ROI gastroenterology dataset.

The mechanism of the hybrid method is to improve the images, segment the affected regions, and extract the feature map through the convolutional layers of the CNN models. The output of the CNN models was fed to PCA to reduce the features, save the most important features, and delete the unnecessary and redundant ones. FFNN and XGBoost receive the most important features and segment them during the training and testing phases. The CNN–FFNN and CNN–XGBoost models can analyze endoscopy images of gastrointestinal diseases and distinguish between the types of gastrointestinal diseases with high accuracy.

Table 3 presents the performance of the hybrid CNN–FFNN for endoscopy image classification of the Kvasir gastroenterology dataset. The GoogLeNet–FFNN achieved an AUC of 93.46%, accuracy of 93.8%, sensitivity of 93.99%, precision of 93.88%, and specificity of 98.91%. The MobileNet–FFNN achieved an AUC of 95.08%, accuracy of 94.4%, sensitivity of 94.45%, precision of 94.49%, and specificity of 98.98%. The DenseNet121–FFNN achieved an AUC of 95.93%, accuracy of 94.8%, sensitivity of 94.76%, precision of 94.84%, and specificity of 99.05%.

Table 4 presents the performance of the hybrid CNN–XGBoost for endoscopy image classification of the Kvasir gastroenterology dataset. The GoogLeNet–XGBoost achieved an AUC of 95.46%, accuracy of 94.3%, sensitivity of 94.29%, precision of 94.26%, and specificity of 99.14%. The MobileNet–XGBoost achieved an AUC of 94.36%, accuracy of 93.8%, sensitivity of 94.06%, precision of 93.83%, and specificity of 99.24%. The DenseNet121–XGBoost achieved an AUC of 94.91%, accuracy of 94.20%, sensitivity of 94.29%, precision of 94.2%, and specificity of 98.95%.

**Figure 6:**
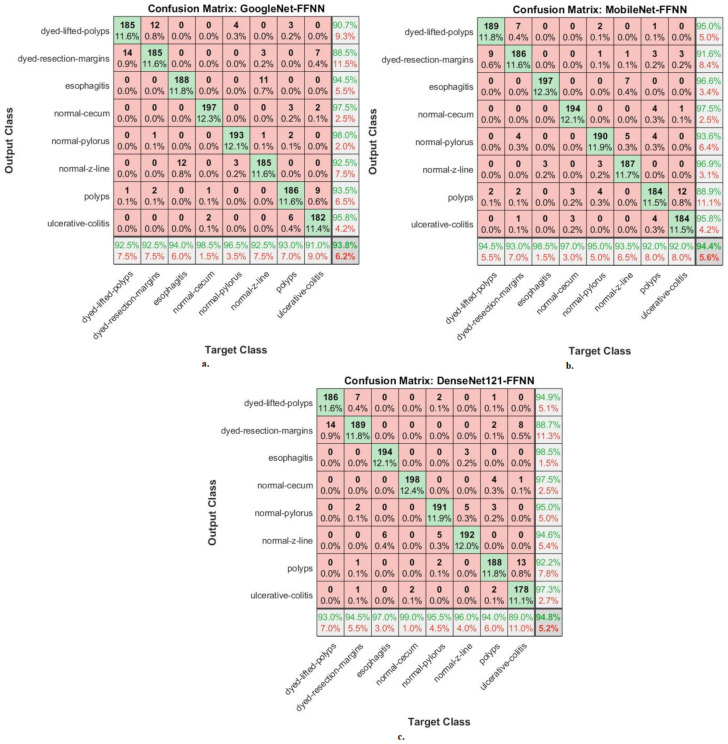
Confusion matrices displaying CNN–FFNN hybrid systems’ performance based on ROIs using the GVF algorithm for endoscopy image analysis of the Kvasir dataset to discriminate gastrointestinal diseases: (**a**) GoogLeNet–FFNN, (**b**) MobileNet–FFNN, and (**c**) DenseNet121–FFNN.

A confusion matrix is the gold standard for presenting system performance measures. This section shows the performance of hybrid CNN–FFNN networks for classifying endoscopy images of the Kvasir-ROI gastroenterology dataset. Figure 6 shows the performance of the GoogLeNet–FFNN, MobileNet–FFNN, and DenseNet121–FFNN hybrid networks through confusion matrices. The hybrid GoogLeNet–FFNN achieved accuracy for each type in the Kvasir gastroenterology dataset as follows: for dyed-lifted-polyps, 92.5%; for dyed resection margins, 92.5%; for esophagitis, 94%, for normal cecum, 98.5%; for normal pylorus, 96.5%; for normal z-line, 92.5%, for polyps, 93%; for ulcerative colitis, 91%. The hybrid MobileNet–FFNN achieved accuracy for each type in the Kvasir gastroenterology dataset as follows: for dyed-lifted-polyps, 94.5%; for dyed resection margins, 93%; for esophagitis, 98.5%; for normal cecum, 97%; for normal pylorus, 95%; for normal z-line, 93.5%; for polyps, 92%; for ulcerative colitis, 92%. The hybrid DenseNet121–FFNN achieved accuracy for each type in the Kvasir gastroenterology dataset as follows: for dyed-lifted-polyps, 93%; for dyed resection margins, 94.5%; for esophagitis, 97%; for normal cecum, 99%; for normal pylorus, 95.5%; for normal z-line, 96%; for polyps, 94%; for ulcerative colitis, 89%.

**Figure 7:**
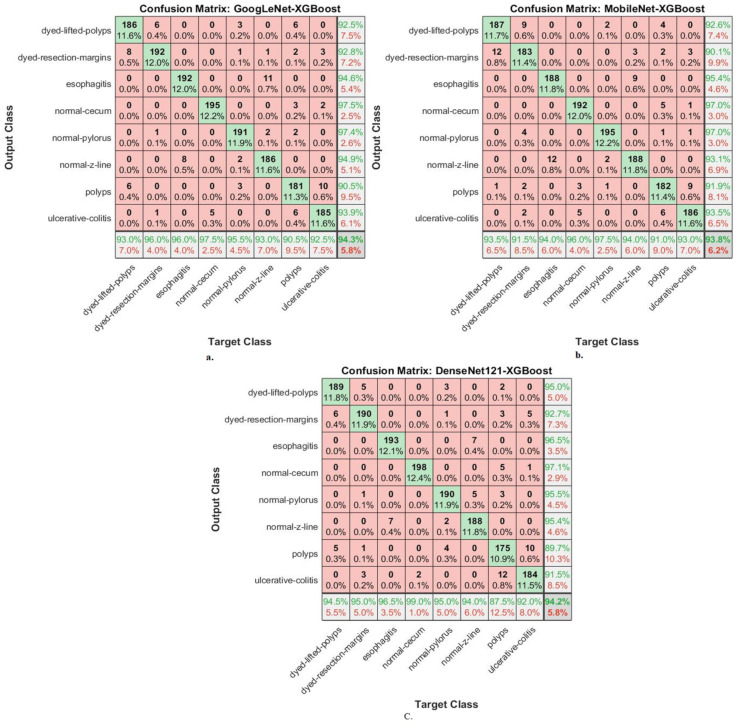
Confusion matrices displaying CNN–XGBoost hybrid systems’ performances based on ROIs using the GVF algorithm for endoscopy image analysis of the Kvasir dataset to discriminate gastrointestinal diseases: (**a**) GoogLeNet–XGBoost, (**b**) MobileNet–XGBoost, and (**c**) DenseNet121–XGBoost.

This section shows the performance of hybrid CNN–XGBoost and CNN–XGBoost networks for classifying endoscopy images from the Kvasir-ROI gastroenterology dataset. Figure 7 shows the performance of the GoogLeNet–XGBoost, MobileNet–XGBoost, and DenseNet121–XGBoost hybrid networks through the confusion matrix. The hybrid GoogLeNet–XGBoost achieved accuracy for each type in the Kvasir gastroenterology dataset as follows: for dyed lifted polyps, 93%; for dyed resection margins, 96%; for esophagitis, 96%; for normal cecum, 97.5%; for normal pylorus, 95.5%; for normal z-line, 93%, for polyps, 90.5%, for ulcerative colitis, 92.5%. The hybrid MobileNet–XGBoost achieved accuracy for each type in the Kvasir gastroenterology dataset as follows: for dyed-lifted-polyps, 93.5%; for dyed resection margins, 91.5%; for esophagitis, 94%; for normal cecum, 96%; for normal pylorus, 97.5%; for normal z-line, 94%; for polyps, 91%; for ulcerative colitis, 93%. The hybrid DenseNet121–XGBoost achieved accuracy for each type in the Kvasir gastroenterology dataset as follows: for dyed lifted polyps, 94.5%; for dyed resection margins, 95%; for esophagitis, 96.5%; for normal cecum, 99%; for normal pylorus, 95%; for normal z-line, 94%; for polyps, 87.5%; for ulcerative colitis, 92%.

### 4.6. Results of Hybrid Methodology Based on Fused Deep Learning Features

The section summarizes the results of the hybrid methodology between the CNN models (GoogLeNet, MobileNet, and DenseNet121) and the FFNN or XGBoost networks based on integrating the features of the CNN models for endoscopy image classification of the Kvasir-ROI gastroenterology dataset. The hybrid methodology aims to optimize the images, segment the affected regions and isolate them from the healthy ones, and extract the feature map through the convolutional layers of the CNN models. The features of the CNN models are sequentially combined as follows: GoogLeNet–MobileNet, MobileNet–DenseNet121, GoogLeNet–DenseNet121, and GoogLeNet–MobileNet–DenseNet121. The features of the hybrid CNN models were then fed into PCA to reduce the features, save the most important features, and delete the unnecessary and redundant features. FFNN and XGBoost receive and breakdown the most important features during the training and testing phases. The CNN-FFNN and CNN-XGBoost, based on features of CNN fusion, analyze endoscopy images of gastroenterology and accurately distinguish between gastroenterological disease types.

Table 5 presents the performance of the hybrid CNN–FFNN based on features of CNN fusion for endoscopy image classification of the Kvasir-ROI gastroenterology dataset. The GoogLeNet–MobileNet–FFNN achieved an AUC of 94.49%, accuracy of 96.06%, sensitivity of 95.75%, precision of 96.09%, and specificity of 99.33%. The MobileNet–DenseNet121–FFNN achieved an AUC of 96.4%, accuracy of 95.8%, sensitivity of 95.64%, precision of 95.84%, and specificity of 99.28%. The GoogLeNet–DenseNet121–FFNN achieved an AUC of 97.21%, accuracy of 96.5%, sensitivity of 96.29%, precision of 96.53%, and specificity of 99.29%. The GoogLeNet–MobileNet–DenseNet121–FFNN achieved an AUC of 97.08%, accuracy of 96.9%, sensitivity of 96.68%, precision of 96.9%, and specificity of 99.3%.

Table 6 presents the performance of the hybrid CNN–XGBoost based on features of CNN fusion for endoscopy image classification of the Kvasir-ROI gastroenterology dataset as follows: The GoogLeNet–MobileNet–XGBoost achieved an AUC of 96.6%, accuracy of 96.8%, sensitivity of 96.43%, precision of 96.78%, and specificity of 99.4%. The MobileNet–DenseNet121–XGBoost achieved an AUC of 95.5%, accuracy of 95.9%, sensitivity of 95.78%, precision of 95.9%, and specificity of 99.23%. The GoogLeNet–DenseNet121–XGBoost achieved an AUC of 95.8%, accuracy of 95.3%, sensitivity of 95.15%, precision of 95.26%, and specificity of 99.15%. The GoogLeNet–MobileNet–DenseNet121–XGBoost achieved an AUC of 97.54%, accuracy of 97.25%, sensitivity of 96.86%, precision of 97.25%, and specificity of 99.48%.

**Figure 8:**
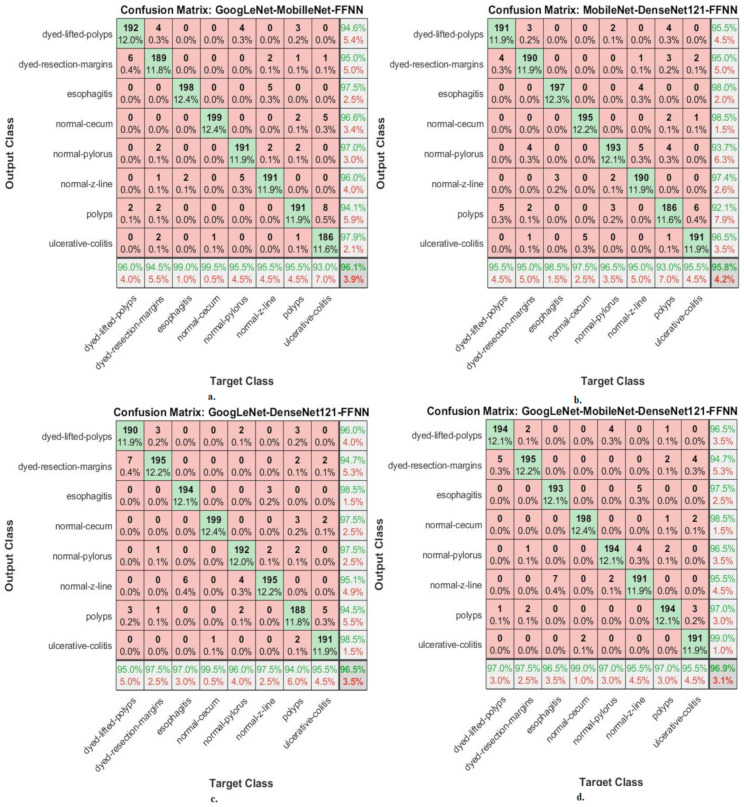
Confusion matrices displaying CNN–FFNN hybrid systems’ performances based on ROIs and feature CNN fused for the endoscopy image analysis of the Kvasir dataset to discriminate gastrointestinal diseases: (**a**) GoogLeNet–MobileNet–FFNN, (**b**) MobileNet–DenseNet121–FFNN, (**c**) GoogLeNet–DenseNet121–FFNN, and (**d**) GoogLeNet–MobileNet–DenseNet121–FFNN.

This section shows the performance of hybrid CNN–FFNN and CNN–XGBoost based on features of CNN fusion for classifying endoscopy images from the Kvasir-ROI gastroenterology dataset. Figure 8 shows the performance of the CNN–FFNN based on the features of CNN fusion through the confusion matrix. The hybrid GoogLeNet–MobileNet–FFNN achieved accuracy for each type in the Kvasir gastroenterology dataset as follows: for dyed lifted polyps, 96%; for dyed resection margins, 94.5%; for esophagitis, 99%; for normal cecum, 99.5%; for normal pylorus, 95.5%; for normal z-line, 95.5%; for polyps, 95.5%; for ulcerative colitis, 93%. The hybrid MobileNet–DenseNet121–FFNN achieved accuracy for each type in the Kvasir gastroenterology dataset as follows: for dyed lifted polyps, 95.5%; for dyed resection margins, 95%; for esophagitis, 98.5%; for normal cecum, 97.5%; for normal pylorus, 96.5%; for normal z-line, 95%; for polyps, 93%; for ulcerative colitis, 95.5%. The hybrid GoogLeNet–DenseNet121–FFNN achieved accuracy for each type in the Kvasir gastroenterology dataset as follows: for dyed lifted polyps, 95%; for dyed resection margin, 97.5%; for esophagitis, 97%; for normal cecum, 99.5%; for normal pylorus, 96%; for normal z-line, 97.5%; for polyps, 94%; for ulcerative colitis, 95.5%. The hybrid GoogLeNet–MobileNet–DenseNet121–FFNN achieved accuracy for each type in the Kvasir gastroenterology dataset as follows: for dyed lifted polyps, 97%; for dyed resection margins, 97.5%; for esophagitis, 96.5%; for normal cecum, 99%; for normal pylorus, 97%; for normal z-line, 95.5%; for polyps, 97%; for ulcerative colitis, 95.5%.

**Figure 9:**
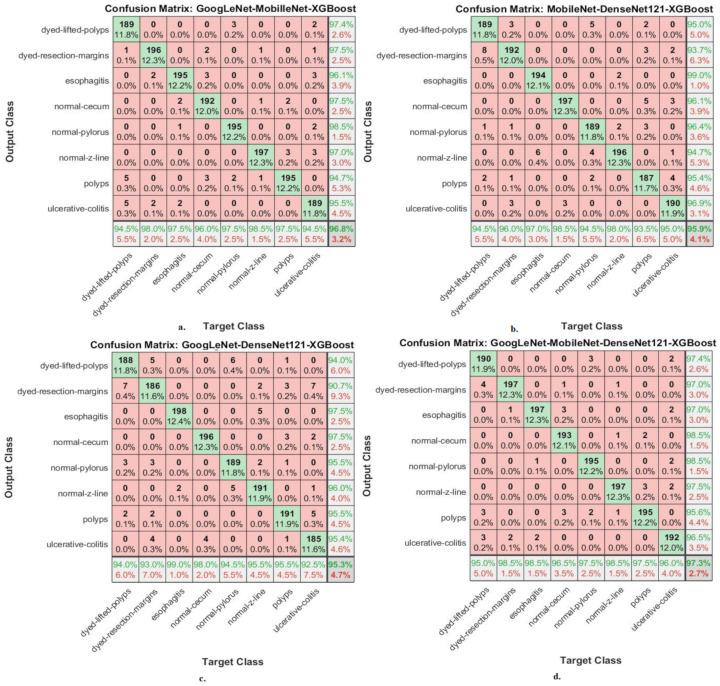
Confusion matrices displaying CNN–XGBoost hybrid systems’ performances based on ROIs and feature CNN fused for the endoscopy image analysis of the Kvasir dataset to discriminate gastrointestinal diseases: (**a**) GoogLeNet–MobileNet–XGBoost, (**b**) MobileNet–DenseNet121–XGBoost, (**c**) GoogLeNet–DenseNet121–XGBoost, and (**d**) GoogLeNet–MobileNet–DenseNet121–XGBoost.

It is noted from Figure 9 that there are cases where the system failed and misclassified the images as follows: First, for the class “dyed-lifted-polyps”, 192 images were classified correctly, whereas the failure cases in this class were 6 images that were classified as “dyed-resection-margins” and 2 images that were classified as “polyps”. Second, for the class “dyed-resection-margins”, 189 images were classified correctly, whereas the failure cases in this class were 4 images that were classified as “dyed-lifted-polyps”, 2 images that were classified as “normal-pylorus”, 1 image that was classified as “normal-z-line”, 2 images that were classified as “polyps”, and 2 images that were classified as “ulcerative-colitis”. Thirdly, for the “esophagitis” class 198 images were classified correctly, whereas the failure cases in this class were 2 images classified as “normal-z-line”. Fourth, for the “normal-cecum” class, 199 images were classified correctly, whereas the failure case in this class was one image that was classified as “ulcerative-colitis”. Fifth, for the “normal-pylorus class”, 191 images were classified correctly, whereas the failure cases in this class were 4 images that were classified as “dyed-lifted-polyps” and 5 images that were classified as “normal-z-line”. Sixth, for the “normal-z-line class”, 191 images were classified correctly, whereas the failure cases in this class were 2 images classified as “dyed-resection-margins”, 5 images classified as “esophagitis”, and 2 images classified as “normal-pylorus”. Seventh, for the “polyps” class, 191 images were classified correctly, whereas the failure cases in this class were 3 images classified as “dyed-lifted-polyps”, 1 image classified as “dyed-resection-margins”, 2 images classified as “normal-cecum”, 2 images classified as “normal-pylorus”, and 1 image classified as “ulcerative-colitis”. Eighth, for the “ulcerative-colitis” class, 186 images were classified correctly, whereas the failure cases in this class were 1 image classified as “dyed-resection-margins”, 5 images classified as “normal-cecum”, and 8 images classified as “polyps”.

Figure 8 shows the performance of the CNN–XGBoost based on features of CNN fusion through the confusion matrix. The hybrid GoogLeNet–MobileNet–XGBoost achieved accuracy for each type in the Kvasir gastroenterology dataset as follows: for dyed lifted polyps, 94.5%; for dyed resection margins, 98%; for esophagitis, 97.5%; for normal cecum, 100%; for normal pylorus, 96%; for normal z-line, 97.5%; for polyps, 98.5%; for ulcerative colitis, 97.5%. The hybrid MobileNet–DenseNet121–XGBoost achieved accuracy for each type in the Kvasir gastroenterology dataset as follows: for dyed lifted polyps, 94.5%; for dyed resection margins, 96%; for esophagitis, 97%; for normal cecum, 98.5%; for normal-pylorus, 94.5%; for normal z-line, 98%; for polyps, 93.5%; for ulcerative colitis, 95%. The hybrid GoogLeNet–DenseNet121–XGBoost achieved accuracy for each type in the Kvasir gastroenterology dataset as follows: for dyed lifted polyps, 94%; for dyed resection margins, 93%; for esophagitis, 99%; for normal cecum, 98%; for normal pylorus, 94.5%; for normal z-line, 95.5%; for polyps, 95.5%; for ulcerative colitis, 92.5%. The hybrid GoogLeNet–MobileNet–DenseNet121–XGBoost achieved accuracy for each type in the Kvasir gastroenterology dataset as follows: for dyed lifted polyps, 95%; for dyed resection margins, 98.5%; for esophagitis, 98.5%; for normal cecum, 96.5%; for normal pylorus, 97.5%; for normal z-line, 98.5%; for polyps, 97.5%; for ulcerative colitis, 96%.

## 5. Discussion of the Performance of the Proposed Methodologies

The gastrointestinal tract suffers from many diseases, such as infections, ulcers, benign tumors, and malignant tumors, which lead to its inability to carry out its tasks well. Additionally, malignant tumors threaten human life and lead to death if discovered late. Polyps may develop into malignancy if not treated and removed early. Manual diagnosis faces many challenges, such as distinguishing between malignant and benign tumors in the early stages and distinguishing between infections and ulcers. Computer-assisted automated diagnostic systems help differentiate between different types of gastrointestinal diseases.

In this study, several hybrid systems (CNN–FFNN and CNN–XGBoost) were developed based on the GVF segmentation algorithm and fused CNN features.

The first methodology was to diagnose endoscopy images from the Kvasir gastroenterology dataset using pre-trained GoogLeNet, MobileNet, and DenseNet121 models, which reached accuracies of 88.2%, 86.4%, and 85.3%, respectively.

The second methodology for diagnosing endoscopy images from the Kvasir gastroenterology dataset was by using pre-trained GoogLeNet, MobileNet, and DenseNet121 models based on the GVF segmentation algorithm and reached accuracies of 90.8%, 91.5%, and 90.1%, respectively.

The third methodology for analyzing endoscopy images for the early diagnosis of gastrointestinal diseases was by hybrid systems (CNN–FFNN and CNN–XGBoost) based on the GVF segmentation algorithm. The GoogLeNet–FFNN, MobileNet–FFNN, and DenseNet121–FFNN hybrid systems achieved accuracies of 93.8%, 94.4%, and 94.8%, respectively, whereas the hybrid systems GoogLeNet–XGBoost, MobileNet–XGBoost, and DenseNet121–XGBoost achieved accuracies of 94.3%, 93.8%, and 94.2%, respectively.

The fourth methodology for the early diagnosis of gastrointestinal diseases was by hybrid systems (CNN–FFNN and CNN–XGBoost) based on the GVF segmentation algorithm and fused CNN features. The hybrid systems GoogLeNet–MobileNet–FFNN, MobileNet–DenseNet121–FFNN, GoogLeNet–DenseNet121–FFNN, and GoogLeNet–MobileNet–DenseNet121–FFNN reached accuracies of 96.06%, 95.8%, 96.5%, and 96.9%, respectively, whereas the hybrid systems GoogLeNet–MobileNet–XGBoost, MobileNet–DenseNet121–XGBoost, GoogLeNet–DenseNet121–XGBoost, and GoogLeNet–MobileNet–DenseNet121–XGBoost reached accuracies of 96.8%, 95.9%, 95.3%, and 97.25%, respectively.

**Figure 10:**
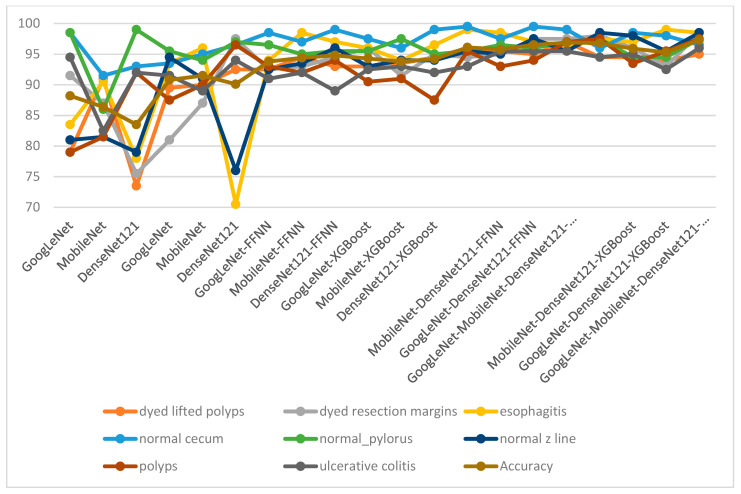
Performance display of all proposed methodologies for analyzing endoscopy images for diagnosing gastroenterological status.

Table 7 and Figure 10 present the results of all proposed methodologies for analyzing endoscopy images for gastroenterology from the Kvasir dataset. The table summarizes the overall accuracy and accuracies for each class in the Kvasir dataset that each methodology yielded. Endoscopic images from the Kvasir gastroenterology dataset were diagnosed using pre-trained GoogLeNet, MobileNet, and DenseNet121 models, which achieved good, but not superior, results. The results of the pre-trained models were improved through the GVF segmentation algorithm, where the images were enhanced and the infected regions were segmented and isolated from the healthy ones. Thus, the pre-trained models received the Kvasir-ROI dataset and an improvement in the results was noted. A hybrid CNN–FFNN or CNN–XGBoost methodology was applied based on the GVF segmentation algorithm, and the results improved significantly. To achieve superior results for the classification of the Kvasir gastroenterology dataset, a hybrid CNN–FFNN or CNN–XGBoost methodology was applied based on the GVF segmentation algorithm and fused CNN features.

The table shows the results for each class across each system and the improvement of the results for each category was as follows: For the class “dyed_lifted_polyps”, the GoogLeNet model achieved an accuracy of 79%, whereas the accuracy was improved to 95.5% by the MobileNet–DenseNet121–FFNN model. For the class “dyed_resection_margins”, the DenseNet121 model achieved an accuracy of 75.5%, whereas the accuracy was improved to 98.5% by the GoogLeNet–MobileNet–DenseNet121–XGBoost model. For the class “esophagitis”, the DenseNet121 model achieved an accuracy of 70.5%, whereas the accuracy was improved to 98.5% by three hybrid models. For the “normal_cecum” class, the MobileNet model achieved an accuracy of 91.5%, whereas the accuracy was improved to 99.5% by the GoogLeNet–MobileNet–FFNN model. For the “normal_pylorus” class, the MobileNet model achieved an accuracy of 86%, whereas the accuracy was improved to 97.5% by the GoogLeNet–MobileNet–DenseNet121–XGBoost model. For the “normal_z_line” class, the DenseNet121 model achieved an accuracy of 76%, whereas the accuracy was improved to 98.5% by the GoogLeNet–MobileNet–XGBoost model. For the “polyps” class, the GoogLeNet model achieved an accuracy of 79%, whereas the accuracy was improved to 98.5% by the GoogLeNet–MobileNet–XGBoost model. For the “ulcerative_colitis” class, the MobileNet model achieved an accuracy of 82.5%, whereas the accuracy was improved to 95.5% by three hybrid models.

It is concluded that the proposed systems are superior to the previous studies by all measures of accuracy, sensitivity, AUC, and specificity thanks to the use of the method of combining CNN features.

Through the results, the following can be concluded: The results of the CNN models based on the GVF algorithm were better than feeding in the images directly without applying the GVF algorithm. The technical results of a hybrid between CNN models (GoogLeNet, MobileNet, and DenseNet121) and FFNN and XGBoost improved the results more than applying CNN models. Finally, it is noted that the performance of the FFNN and XGBoost networks with the hybrid features of the CNN models is clearly better than the previous methods.

## 6. Conclusions

It is difficult to distinguish between the types of diseases of gastrointestinal tract and the presence of anatomical landmarks, pathological findings, and polyp removal, especially in the early stages. Therefore, many methodologies have been developed to help doctors and specialists diagnose disease from endoscopy images of gastrointestinal tract. The Kvasir dataset was classified by three pre-trained CNN models. In the second methodology, endoscopy images were optimized, and the affected areas were segmented and classified by three pre-trained CNN models. The third methodology analyzed endoscopy images from the Kvasir dataset by a hybrid CNN–FFNN or CNN–XGBoost methodology based on the GVF segmentation algorithm. The fourth methodology analyzed endoscopy images from the Kvasir dataset by a hybrid CNN–FFNN or CNN–XGBoost methodology based on the GVF segmentation algorithm and fused CNN features. The GoogLeNet–MobileNet–DenseNet121–XGBoost hybrid methodology based on the GVF algorithm achieved an AUC of 97.54%, accuracy of 97.25%, sensitivity of 96.86%, precision of 97.25%, and specificity of 99.48%.

These systems can be generalized to help doctors and endoscopy specialists in the early diagnosis of tumors of the gastrointestinal tract in order for the patient to receive appropriate treatment.

The most important limitation we encountered was the similarity in the biological characteristics between some types of gastrointestinal diseases, which were overcome by combining the features of the CNN models.

The future work is to extract the handcrafted features, combine them with combined CNN features, and generalize them to the proposed systems on a new dataset.

Additionally, in future work PCA will be used before and after combining the features and comparing the results.

## Figures and Tables

**Table 1:** Results of pre-trained deep learning models for endoscopy image analysis of the Kvasir dataset to discriminate gastrointestinal diseases.

Models	Type of Disease	AUC %	Accuracy %	Sensitivity %	Precision %	Specificity %
GoogLeNet	dyed_lifted_polyps	88.7	79	79.2	91.3	99.2
	dyed_resection_margins	92.5	91.5	92.4	83.2	96.5
	esophagitis	90.2	83.5	83.1	82.7	97.4
	normal_cecum	94.5	98.5	97.6	91.6	98.7
	normal_pylorus	93.8	98.5	97.8	92.9	99.3
	normal_z_line	91.6	81	81.2	81.8	96.9
	polyps	86.2	79	78.9	94	98.6
	ulcerative_colitis	94.2	94.5	94.2	89.2	98.9
	**Average ratio**	**91.46**	**88.20**	**88.05**	**88.34**	**98.19**
MobileNet	dyed_lifted_polyps	92.6	91	91.2	88.8	98.1
	dyed_resection_margins	90.4	87	87.3	87.4	98.3
	esophagitis	92.1	90.5	90.8	89.2	97.6
	normal_cecum	93.2	91.5	91.5	91.5	99.4
	normal_pylorus	95.4	96	85.7	86.4	98.8
	normal_z_line	88.1	81.5	80.9	83.6	97.5
	polyps	86.4	81.5	81.2	76.9	96.3
	ulcerative_colitis	89.7	82.5	82.1	88.2	97.7
	**Average ratio**	**90.99**	**86.40**	**86.34**	**86.50**	**97.96**
DenseNet121	dyed_lifted_polyps	82.4	73.5	72.9	75.4	97.4
	dyed_resection_margins	86.2	75.5	76.2	77.8	96.6
	esophagitis	81.2	78	78.4	78.4	96.9
	normal_cecum	94.6	93	92.8	95.4	98.6
	normal_pylorus	95.2	99	98.7	97.1	99.6
	normal_z_line	83.4	79	78.8	77.5	96.7
	polyps	94.9	92	91.7	84.4	97.8
	ulcerative_colitis	95.1	92	92.3	96.3	99.2
	**Average ratio**	**89.13**	**85.30**	**85.23**	**85.29**	**97.85**

**Table 2:** Results of CNN models based on the GVF Algorithm for endoscopy image analysis of the Kvasir dataset to discriminate gastrointestinal diseases.

Models	Type of Disease	AUC %	Accuracy %	Sensitivity %	Precision %	Specificity %
GoogLeNet	dyed_lifted_polyps	91.2	89.5	90.2	85.2	98.2
	dyed_resection_margins	88.3	81	80.8	87.1	97.8
	esophagitis	95.1	93.5	93.7	95.4	99.3
	normal_cecum	92.3	93.5	94.3	94.4	99.4
	normal_pylorus	93.1	95.5	95.2	95.5	98.7
	normal_z_line	92.9	94.5	93.8	92.6	99.1
	polyps	89.1	87.5	88.4	87.9	97.6
	ulcerative_colitis	92.4	91.5	92.3	88.4	97.8
	**Average ratio**	**91.80**	**90.80**	**91.09**	**90.81**	**98.49**
MobileNet	dyed_lifted_polyps	91.2	90	90.4	90.9	98.5
	dyed_resection_margins	89.3	87	86.9	89.7	99.1
	esophagitis	92.4	96	95.6	93.2	98.6
	normal_cecum	93.1	95	95.1	95	99.1
	normal_pylorus	92.6	94	93.8	93.5	99.4
	normal_z_line	93.4	91	91.1	93.8	98.8
	polyps	90.9	90	90.4	84.1	97.9
	ulcerative_colitis	91.8	89	89.2	92.2	99.2
	**Average ratio**	**91.84**	**91.50**	**91.56**	**91.55**	**98.83**
DenseNet121	dyed_lifted_polyps	91.3	92.5	93.2	89.8	98.2
	dyed_resection_margins	94.2	97.5	96.8	95.1	99.1
	esophagitis	80.2	70.5	70	70.5	95.6
	normal_cecum	94.3	96.5	96.2	95.5	98.7
	normal_pylorus	95.2	97	97.3	97.5	99.5
	normal_z_line	83.4	76	75.6	78.4	96.7
	polyps	94.9	96.5	95.8	96	99.2
	ulcerative_colitis	93.6	94	93.7	97.4	99.8
	**Average ratio**	**90.89**	**90.10**	**89.83**	**90.03**	**98.35**

**Table 3:** Results of CNN–FFNN hybrid system based on ROI using the GVF algorithm for endoscopy image analysis of the Kvasir dataset to discriminate gastrointestinal diseases.

Hybrid Models	Type of Lesion	AUC %	Accuracy %	Sensitivity %	Precision %	Specificity %
GoogLeNet–FFNN	dyed_lifted_polyps	92.5	92.5	93.2	90.7	99.2
	dyed_resection_margins	91.7	92.5	92.8	88.5	97.6
	esophagitis	93.6	94	94.4	94.5	98.7
	normal_cecum	96.8	98	97.8	97.5	99.8
	normal_pylorus	95.7	96.5	96.3	98	99.5
	normal_z_line	92.4	92.5	92.9	92.5	98.6
	polyps	92.9	93	93.3	93.5	99.1
	ulcerative_colitis	92.1	91	91.2	95.8	98.8
	**Average ratio**	**93.46**	**93.80**	**93.99**	**93.88**	**98.91**
MobileNet–FFNN	dyed_lifted_polyps	95.4	94.5	94.2	95	98.7
	dyed_resection_margins	94.6	93	93.4	91.6	99.4
	esophagitis	97.5	98.5	98.1	96.6	99.1
	normal_cecum	96.8	97	97.2	97.5	99.5
	normal_pylorus	96.3	95	94.8	93.6	98.5
	normal_z_line	94.1	93.5	93.9	96.9	99.6
	polyps	92.8	92	92.4	88.9	97.8
	ulcerative_colitis	93.1	92	91.6	95.8	99.2
	**Average ratio**	**95.08**	**94.40**	**94.45**	**94.49**	**98.98**
DenseNet121–FFNN	dyed_lifted_polyps	94.1	93	92.7	94.9	98.7
	dyed_resection_margins	96.3	94.5	94.2	88.7	97.6
	esophagitis	97.6	97	97.3	98.5	99.5
	normal_cecum	98.2	99	99.1	97.5	99.7
	normal_pylorus	96.1	95.5	95.2	95	98.6
	normal_z_line	97.2	96	96.4	94.6	99.4
	polyps	95.8	94	93.9	92.2	99.1
	ulcerative_colitis	92.1	89	89.3	97.3	99.8
	**Average ratio**	**95.93**	**94.80**	**94.76**	**94.84**	**99.05**

**Table 4:** Results of CNN–XGBoost hybrid system based on ROI using the GVF algorithm for endoscopy image analysis of the Kvasir dataset to discriminate gastrointestinal diseases.

Hybrid Models	Type of Disease	AUC %	Accuracy %	Sensitivity %	Precision %	Specificity %
GoogLeNet–XGBoost	dyed_lifted_polyps	93.8	93	93.2	92.5	99.2
	dyed_resection_margins	96.5	96	96.4	92.8	99.4
	esophagitis	97.3	96	95.8	94.6	98.7
	normal_cecum	98.6	97.5	97.2	97.5	99.5
	normal_pylorus	96.2	95.5	94.7	97.4	99.8
	normal_z_line	94.9	93	93.4	94.9	98.6
	polyps	92.3	90.5	90.9	90.5	99.4
	ulcerative_colitis	94.1	92.5	92.7	93.9	98.5
	**Average ratio**	**95.46**	**94.30**	**94.29**	**94.26**	**99.14**
MobileNet– XGBoost	dyed_lifted_polyps	94.1	93.5	94.1	92.6	99.2
	dyed_resection_margins	92.4	91.5	92.4	90.1	98.8
	esophagitis	93.9	94	94.4	95.4	99.3
	normal_cecum	95.7	96	95.6	97	99.5
	normal_pylorus	98.4	97.5	97.3	97	99.7
	normal_z_line	94.7	94	94.2	93.1	98.9
	polyps	92.1	91	91.1	91.9	99.1
	ulcerative_colitis	93.6	93	93.4	93.5	99.4
	**Average ratio**	**94.36**	**93.80**	**94.06**	**93.83**	**99.24**
DenseNet121–XGBoost	dyed_lifted_polyps	93.8	94.5	94.2	95	98.5
	dyed_resection_margins	94.2	95	95.4	92.7	99.1
	esophagitis	96.9	96.5	95.9	96.5	98.8
	normal_cecum	98.2	99	99.1	97.1	99.5
	normal_pylorus	95.3	95	95.4	95.5	98.6
	normal_z_line	94.9	94	93.8	95.4	98.8
	polyps	92.8	87.5	88.4	89.7	99.4
	ulcerative_colitis	93.2	92	92.1	91.5	98.9
	**Average ratio**	**94.91**	**94.20**	**94.29**	**94.20**	**98.95**

**Table 5:** Results of CNN–FFNN hybrid system based on feature CNN fused for endoscopy image analysis of the Kvasir dataset to discriminate gastrointestinal diseases.

Fusion Features	Classifier	Type of Disease	AUC %	Accuracy %	Sensitivity %	Precision %	Specificity %
GoogLeNet–MobileNet	FFNN	dyed_lifted_polyps	95.9	96	96.2	94.6	99.2
		dyed_resection_margins	94.2	94.5	94.4	95	99.4
		esophagitis	98.7	99	98.6	97.5	99.8
		normal_cecum	99.1	99.5	99.1	96.6	98.8
		normal_pylorus	96.4	95.5	94.6	97	99.5
		normal_z_line	96.7	95.5	94.9	96	98.6
		polyps	96.1	95.5	95.4	94.1	99.4
		ulcerative_colitis	94.8	93	92.8	97.9	99.9
		**Average ratio**	**96.49**	**96.06**	**95.75**	**96.09**	**99.33**
MobileNet–DenseNet121	FFNN	dyed_lifted_polyps	96.2	95.5	95.2	95.5	98.5
		dyed_resection_margins	95.7	95	94.8	95	99.2
		esophagitis	98.5	98.5	98.4	98	99.9
		normal_cecum	98.1	97.5	97.1	98.5	99.6
		normal_pylorus	96.4	96.5	95.8	93.7	98.7
		normal_z_line	95.9	95	95.4	97.4	99.8
		polyps	94.1	93	93.2	92.1	99.1
		ulcerative_colitis	96.3	95.5	95.2	96.5	99.4
		**Average ratio**	**96.40**	**95.80**	**95.64**	**95.84**	**99.28**
GoogLeNet–DenseNet121	FFNN	dyed_lifted_polyps	96.2	95	95.2	96	98.5
		dyed_resection_margins	97.9	97.5	97.1	94.7	99.2
		esophagitis	96.8	97	96.8	98.5	99.8
		normal_cecum	99.1	99.5	99.3	97.5	99.7
		normal_pylorus	97.2	96	95.9	97.5	99.5
		normal_z_line	98.4	97.5	96.6	95.1	99.2
		polyps	95.6	94	94.3	94.4	98.6
		ulcerative_colitis	96.5	95.5	95.1	98.5	99.8
		**Average ratio**	**97.21**	**96.50**	**96.29**	**96.53**	**99.29**
GoogLeNet–MobileNet–DenseNet121	FFNN	dyed_lifted_polyps	97.8	97	97.2	96.5	99.2
		dyed_resection_margins	98.2	97.5	97.4	94.7	98.7
		esophagitis	97.1	96.5	95.8	97.5	99.5
		normal_cecum	98.9	99	99.1	98.5	99.7
		normal_pylorus	97.5	97	96.6	96.5	98.8
		normal_z_line	95.1	95.5	94.8	95.5	99.1
		polyps	96.2	97	96.9	97	99.6
		ulcerative_colitis	95.8	95.5	95.6	99	99.8
		**Average ratio**	**97.08**	**96.90**	**96.68**	**96.90**	**99.30**

**Table 6:** Results of CNN–XGBoost hybrid system based on feature CNN fused for endoscopy image analysis of the Kvasir dataset to discriminate gastrointestinal diseases.

Fusion Features	Classifier	Type of Disease	AUC %	Accuracy %	Sensitivity %	Precision %	Specificity %
GoogLeNet–MobileNet	XGBoost	dyed_lifted_polyps	95.1	94.5	94.2	97.4	99.5
		dyed_resection_margins	97.8	98	98.1	97.5	99.8
		esophagitis	96.8	97.5	96.7	96.1	98.5
		normal_cecum	95.7	96	96.4	97.5	100
		normal_pylorus	96.2	97.5	97.3	98.5	99.6
		normal_z_line	98.8	98.5	97.8	97	99.8
		polyps	97.2	97.5	96.5	94.7	98.8
		ulcerative_colitis	95.2	94.5	94.4	95.5	99.2
		**Average ratio**	**96.60**	**96.80**	**96.43**	**96.78**	**99.40**
MobileNet–DenseNet121	XGBoost	dyed_lifted_polyps	93.2	94.5	93.8	95	99.2
		dyed_resection_margins	95.1	96	96.2	93.7	98.7
		esophagitis	98.1	97	96.9	99	99.6
		normal_cecum	97.2	98.5	98.4	96.1	98.9
		normal_pylorus	93.8	94.5	93.7	96.4	99.4
		normal_z_line	96.8	98	97.7	94.7	98.8
		polyps	95.6	93.5	94.1	95.4	99.4
		ulcerative_colitis	94.2	95	95.4	96.9	99.8
		**Average ratio**	**95.5**	**95.9**	**95.78**	**95.9**	**99.23**
GoogLeNet–DenseNet121	XGBoost	dyed_lifted_polyps	94.2	94	94.2	94	99.4
		dyed_resection_margins	93.9	93	93.4	90.7	98.8
		esophagitis	99.4	99	98.7	97.5	99.6
		normal_cecum	97.8	98	97.6	97.5	100
		normal_pylorus	95.4	94.5	94.3	95.5	98.5
		normal_z_line	95.9	95.5	94.9	96	99.2
		polyps	96.1	95.5	95.3	95.5	99.4
		ulcerative_colitis	93.7	92.5	92.8	95.4	98.3
		**Average ratio**	**95.80**	**95.30**	**95.15**	**95.26**	**99.15**
GoogLeNet–MobileNet–DenseNet121	XGBoost	dyed_lifted_polyps	94.6	95	94.6	97.4	99.5
		dyed_resection_margins	98.6	98.5	97.8	97	99.7
		esophagitis	98.4	98.5	98.2	97	99.6
		normal_cecum	97.1	96.5	95.9	98.5	100
		normal_pylorus	98.1	97.5	97.4	98.5	99.7
		normal_z_line	99.2	98.5	98.2	97.5	99.9
		polyps	98.7	97.5	97.3	95.6	98.8
		ulcerative_colitis	95.6	96	95.5	96.5	98.6
		**Average ratio**	**97.54**	**97.25**	**96.86**	**97.25**	**99.48**

**Table 7:** Results of proposed methodologies for analyzing endoscopy images for early diagnosis of gastroenterological status from the Kvasir dataset.

Techniques		Features	Dyed Lifted Polyps	Dyed Resection Margins	Esophagitis	Normal Cecum	Normal Pylorus	Normal z Line	Polyps	Ulcerative Colitis	Accuracy
pre-trained	GoogLeNet		79	91.5	83.5	98.5	98.5	81	79	94.5	88.2
	MobileNet		91	87	90.5	91.5	86	81.5	81.5	82.5	
	DenseNet121		73.5	75.5	78	93	99	79	92	92	83.5
Based on GVF	GoogLeNet		89.5	81	93.5	93.5	95.5	94.5	87.5	91.5	90.8
	MobileNet		90	87	96	95	94	91	90	89	91.5
	DenseNet121		92.5	97.5	70.5	96.5	97	76	96.5	94	90.1
Hybrid	FFNN	GoogLeNet	92.5	92.5	94	98.5	96.5	92.5	93	91	93.8
		MobileNet	94.5	93	98.5	97	95	93.5	92	92	94.4
		DenseNet121	93	94.5	97	99	95.5	96	94	89	94.8
Hybrid	XGBoost	GoogLeNet	93	96	96	97.5	95.5	93	90.5	92.5	94.3
		MobileNet	93.5	91.5	94	96	97.5	94	91	93	93.8
		DenseNet121	94.5	95	96.5	99	95	94	87.5	92	94.2
Hybrid	FFNN	GoogLeNet–MobileNet	96	94.5	99	99.5	95.5	95.5	95.5	93	96.1
		MobileNet–DenseNet121	95.5	95	98.5	97.5	96.5	95	93	95.5	95.8
		GoogLeNet–DenseNet121	95	97.5	97	99.5	96	97.5	94	95.5	96.5
		GoogLeNet–MobileNet–DenseNet121	97	97.5	96.5	99	97	95.5	97	95.5	96.9
Hybrid	XGBoost	GoogLeNet–MobileNet	94.5	98	97.5	96	97.5	98.5	97.5	94.5	96.8
		MobileNet–DenseNet121	94.5	96	97	98.5	94.5	98	93.5	95	95.9
		GoogLeNet–DenseNet121	94	93	99	98	94.5	95.5	95.5	92.5	95.3
		GoogLeNet–MobileNet–DenseNet121	95	98.5	98.5	96.5	97.5	98.5	97.5	96	97.3

## Data Availability

In this study, data supporting the performance of the proposed methodologies were collected from the publicly available Kvasir gastroenterology dataset at the link: https://datasets.simula.no/kvasir/#download (accessed date: 2 October 2022).
